# Flag-waving behavior in matador bugs is an antipredatory strategy

**DOI:** 10.1093/cz/zoaf047

**Published:** 2025-08-01

**Authors:** Connor Evans-Blake, Juliette J Rubin, Ummat Somjee

**Affiliations:** Department of Earth and Environmental Sciences, University of Manchester, Oxford Rd, Manchester M13 9PL, UK; Smithsonian Tropical Research Institute, Panama City 0843-03092, Panamá; Smithsonian Tropical Research Institute, Panama City 0843-03092, Panamá; Department of Integrative Biology, University of Texas at Austin, Austin, TX 78712, USA

**Keywords:** antipredator behavior, behavioral plasticity, *Bitta alipes*, conspicuous signaling, Coreidae, facultative defense

## Abstract

To dissuade predator attack, animals may advertise their chemical defense with bright coloration and specialized behaviors. However, these antipredator adaptations can be costly, if they unnecessarily draw attention to the prey animal. Thus, animals with elaborate antipredator traits may be under pressure to selectively increase advertisement of their defenses in particular contexts. The matador bug, *Bitta alipes* (Hemiptera: Coreidae), possesses large, colorful flags on its hind tibia that it uses in a stereotypic “waving” behavior. Previous research found no evidence that this waving behavior is employed in social or sexual interactions. Here, we experimentally tested for a potential antipredator function of flag waving by exposing a matador bug to either an arthropod predator (praying mantid) or a similarly sized nonpredatory arthropod (nonpredatory katydid). In total, we recorded 2,938 leg waves among 25 individuals. We found that matador bugs’ waving behavior increased in duration, frequency (number of wave bouts), and intensity (number of waves per bout) in the presence of praying mantids. Notably, we found on average a 7-fold increase in the number of waves in the presence of a mantid relative to a similarly sized nonpredatory arthropod. Praying mantids consumed very few matador bugs (3/25) and never attacked bugs that were waving, lending support to the hypothesis that flags serve an antipredator function in matador bugs. We find similar flag-waving behavior in at least 5 closely-related flag-legged bug species, all of which have expanded tibial flags with contrasting coloration and are *Passiflora* specialists, providing opportunities for future studies to examine the evolution of elaborate flag-waving behavior as an antipredator behavior in this group.

## Graphical Abstract

**Graphical Abstract:**
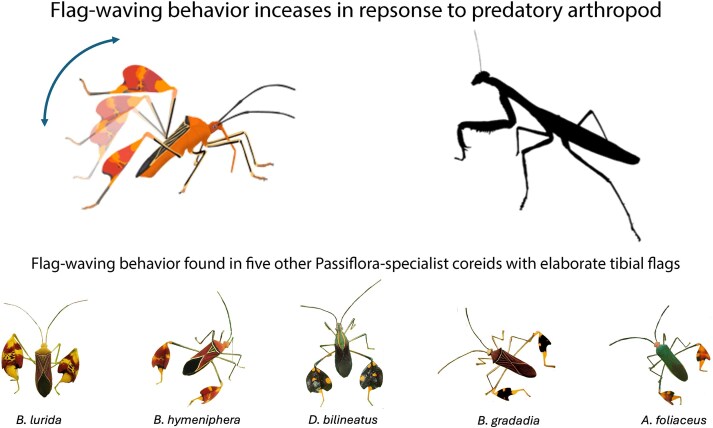

Many animals have evolved elaborate traits and behaviors that serve antipredation functions (Eisner et al. 2005). Conspicuous traits, such as bright coloration, enlarged appendages, and sound-producing structures can serve multiple functions in predator deterrence and avoidance (Wang and Shaffer 2008; Conner and Corcoran 2012; Sourakov 2013; Rubin et al. 2018). Some conspicuous traits are most effective when paired with specific antipredator behaviors, such as the tails on lycaenid butterfly hindwings that, in combination with hindwing movements, can create a false-head appearance (Wourms and Wasserman 1985) or the displays of highly elaborated femora in boxer mantids, which reduce predatory attacks (Li et al. 2025). Another example comes from brightly colored wings of some katydid species that, when revealed during escape maneuvers, can create deimatic startle displays (Nickle and Castner 1995; Drinkwater et al. 2022). The question of when to deploy a conspicuous antipredator trait can be a crucial one, as early or unnecessary deployment may be costly (in energetic or time loss) and could call unwanted attention to an otherwise undetected prey animal (Edmunds 1974). Thus, the ability to detect threats in the environment and perform a targeted response in the context of true danger can be under strong selection.

The ability for selective antipredator response has been well documented in vertebrates: rattlesnakes deploy their rattle when predators are near (Allf et al. 2016), broad-headed skinks undulate their colorful tail on approach of a human predator (Cooper 1998), and motmots wag their racqueted tails as a pursuit-deterrent signal to nearby predators (Murphy 2006). However, specifically targeted, facultatively deployed antipredator responses to predator presence have been less clearly identified in insects. That is, although there are many examples of deimatic displays in insects, most of these are elicited by generalized tactile stimulation and are not necessarily predator-specific (Vidal-García et al. 2020; Drinkwater et al. 2022). Examples of insects parsing predator cues from other stimuli and responding with specialized antipredator behaviors or morphological traits are rarer. For example, although it has been hypothesized that lycaenid false head behaviors are deployed predominantly in the presence of predators, trials between a stuffed-bird predatory threat and lycaenid butterflies reveals equivocal evidence that they increase waving of their false antennae in response to this perceived predator threat (López-Palafox et al. 2015).

A nonmutually exclusive alternative to dissuading predator attack occurs through deflection, where prey evolve traits and behaviors that influence the position of body the predator strikes, which may enhance the likelihood of prey surviving an attack (Hill and Vaca 2004; Barber et al. 2015; Humphreys and Ruxton 2018; Rubin et al. 2018). Organisms in nature often face different types of predators with different sensory systems, which may lead to the evolution of multiple antipredator adaptations (Kikuchi et al. 2023). For example, obligate signaling, such as aposematic coloration in long-wavelength colors (e.g., reds and yellows), may be effective against tetrachromatic predators like birds, but may not be effective against mono- or dichromatic arthropod predators, such as praying mantids (Fabricant and Herberstein 2015). Thus, organisms may use obligate signals alongside facultative signaling to enhance communication directed toward different types of predators (Rowe 1999; Hebets and Papaj 2005; Mappes et al. 2005; Stevens 2013; Humphreys and Ruxton 2018). Thus, multiple antipredation traits likely often function together, creating a layered defense, which may allow traits to be effective against multiple predators (Hebets and Papaj 2005; Pegram and Rutowski 2014; Humphreys and Ruxton 2018; Rojas et al. 2018; Kikuchi et al. 2023).

One example of an insect with layered obligate and facultative aposematic signals is the matador bug, *Bitta alipes* (= *Anisoscelis alipes*; Hemiptera: Coreidae) (Leavengood et al. 2024). These bugs and several of their sister species display conspicuous, highly elaboratated hind tibia expansions whose size can be comparable to, or exceed, the width of the pronotum (Longbottom et al. 2022, Fig. 1). Flags are present and equally sized (when accounting for body size) in both sexes (Longbottom et al. 2022). They are also used in a waving behavior, where an individual will raise either one or both hind legs above the body and often perform oscillatory movements of the tibial flags by rotating the tibia independently of the femur, while moving the femur up and down (see Supplementary S3, Video S1). This behavior appears to be selectively performed at specific times, but an experimental study revealed no evidence that waving has a social or sexual function. That is, there were no significant differences between males and females in the frequency, rate, or time spent performing waving behavior, nor was the sex of the surrounding conspecifics predictive of number or waves or rate of waving behavior (Longbottom et al. 2022). A separate study, testing the function of these flags against wild-caught motmot bird predators, revealed that these flags and overall aposematic coloring of the bug signal unpalability to avian predators (Rubin et al. 2024). This study did not specifically test the waving behavior of the matador bugs in a predation context, however. Thus, the major selective forces that shape this elaborate waving behavior remains unknown.

**Figure 1:**
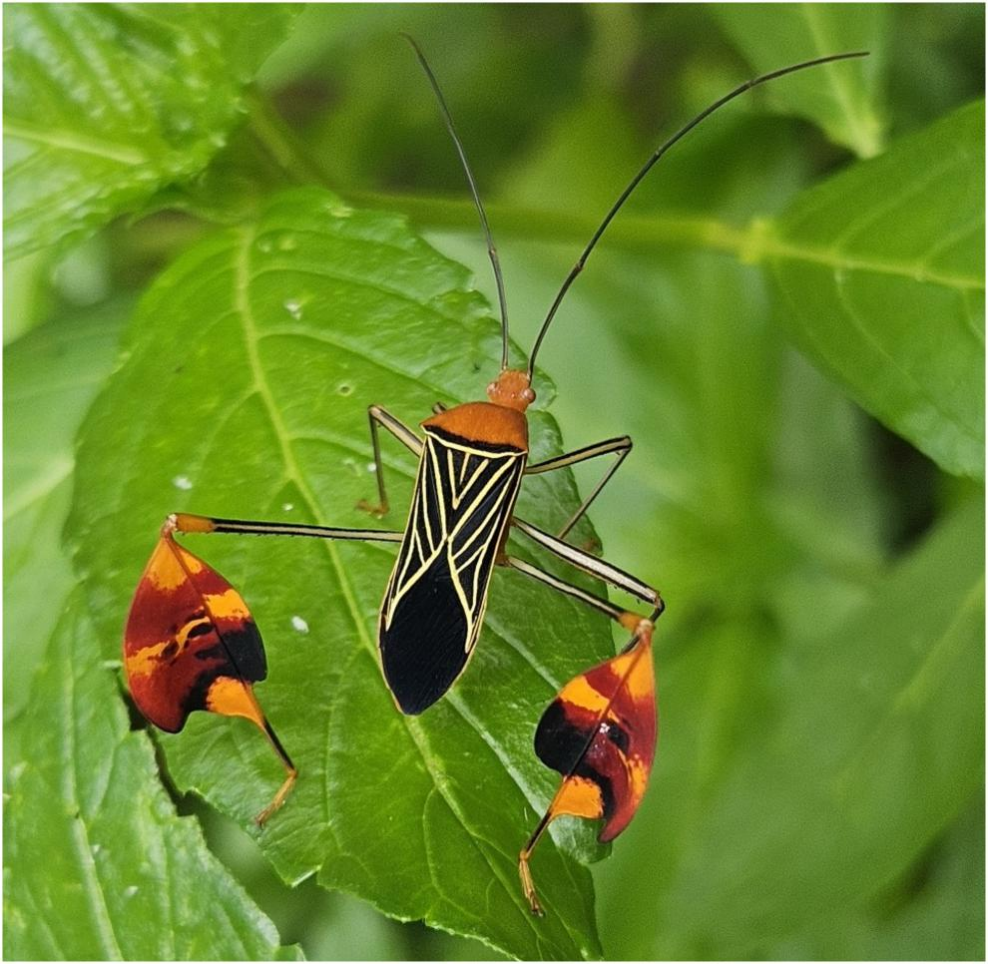
Matador bug, *Bitta alipes* (= *Anisoscelis alipes*), possesses large, conspicuous tibial expansions on its hind legs, referred to as “flags,” with which they perform a “waving” behavior. These traits play a role in antipredation strategies (Photo:© Alejandra Vega, iNatularist, Colombia, 2024, https://www.inaturalist.org/observations/224203031, License: CC BY-NC 4.0).

Given the lack of evidence for a social function of flag waving, and given that bird predators are cuing in to the flags as an aposematic signal, we hypothesized that flag waving is an antipredator trait specifically targeted at smaller, close-range predators, such as arthropods. We therefore predicted that aspects of flag-waving behavior (frequency, intensity, duration) of the matador bug would increase in response to the presence of a predatory arthropod compared with a nonpredatory arthropod. To test this, we placed matador bugs in a plexiglass enclosure with either a sympatric praying mantid (Mantodea; predator) or a sympatric katydid (Orthoptera; nonpredator). We chose praying mantids as our representative predators because of their opportunistic hunting behavior (Corrette 1990), broad diets (Fagan et al. 2002), and documented observations of preying on matador bugs (Somjee U, personal observation). Praying mantids are visual predators whose visual system utilizes stereoscopic cues to detect and respond to prey based on visual features such as movement and size (Rilling et al. 1959; Paradise and Stamp 1991; Nityananda et al. 2018). Their eyes are monochromatic, possessing one color receptor that is likely not very sensitive to long-wavelength colors (e.g., reds and yellows) that are typically used in aposematic coloration schema for some vertebrate predators (Fabricant and Herberstein 2015). We chose a katydid as our nonpredatory arthropod control, as they are primarily herbivorous (Gwynne 2008; Palmer et al. 2022), and co-occur in similar habitats as both mantids and matador bugs. In addition to our experimental study, we conducted a survey of recorded videos online of closely related coreid bugs in the tribe to assess the commonality of waving behavior in closely related bugs with similar morphologies. Together, this study contributes to our understanding of the elaborate morphology and waving behavior of this little-studied group.

## Materials and methods

### Insect collection and rearing

Using a sweep net, we collected *Bitta alipes* (Hemiptera: Coreidae: Aniscocelinidi) from *Passiflora vitifolia* around Gamboa, Panamá. Immediately after collection, the insects were placed in a butterfly house (45 × 45 × 100 cm) before being transferred to a larger insect house (500 × 300 × 200 cm) for rearing, located in the insectaries at the Smithsonian Tropical Research Institute (STRI) in Gamboa, Panamá. This larger insect house contained various potted plant species (Family: Passifloraceae), which were watered daily to create an environment that supported high survival and breeding rates for the insects (Longbottom et al. 2022). The house was equipped with feeding plates refreshed with frozen maracuyá (*Passiflora flavicarpa*) every 2 days to allow for ad libitum feeding. We regularly replaced the plants with fresh ones to facilitate leaf regrowth, as the matador bugs also fed on them.

Praying mantids (Family: Mantidae) from 3 genera (*Stagmatoptera*, *Stagmomantis* and *Parastagmatoptera*) and katydids (Family: Tettigoniidae) from 3 genera (*Philophyllia*, *Microcentrum* and *Itarissa*) were collected by hand around Gamboa, Panamá. After collection, insects were housed individually in butterfly cages (45 × 45 × 100 cm), each accompanied by a paper towel that was dampened every 2 days and provided with a stick to allow for a natural resting position. We kept insects at ambient temperature and humidity to mimic natural conditions. Each praying mantid was fed a live cricket every 2 days, except for a 3-day fasting period prior to the start of experiments to increase motivation during the trials. We measured the body size of the praying mantids and katydids by recording the width of their pronotum on collection.

### Predator treatments

All trials were conducted in a shaded area in the ambient temperature and humidity of Gamboa, Panamá. To assess the effect of a praying mantid on flag-waving behavior, individual matador bugs were exposed to 2 treatments: 1) predator (praying mantid) and 2) nonpredator (katydid). Each trial took place in a plexiglass cube (15 × 15 × 15 cm) containing a twig to facilitate more natural insect movement (Supplementary Fig. S1). Prior to trial start, we measured the pronotum width of each matador bug, recorded its sex, and marked each individual with a unique number using nontoxic, fast-drying paint applied as a small marking on the pronotum to facilitate identification. The marking was positioned in a way that it was unlikely to fall within the predator's visual range, and it was designed to avoid interfering with the bug's waving behavior or its ability to perceive and respond to potential threats. Each individual bug (*n* = 25) was then placed in the plexiglass cube for an initial no-predator treatment to allow for acclimation. Although this period primarily served as acclimation, we also recorded the frequency of waving behavior to establish baseline activity levels in the absence of any stimulus. A total of 7 praying mantids and 3 katydids were used in the experiment, with each matador bug exposed to 1 mantid individual and 1 katydid individual in separate trials. Thus, each of our 25 bugs (*n*_bugs_ = 25) had a recorded baseline, predator exposure (mantid), and nonpredator exposure (mantid). We first conducted a set of trials where the katydid was introduced first (*n*_trial_ = 9), followed by a set where the mantid was introduced first (*n*_trial_ = 9), and finally a set where the first treatment was randomized (*n*_trial_ = 7). This ensured that each predator was used an equal number of times across trials. Trial set was incorporated into our statistical model to account for the nonrandom order of the first 2 sets of trials. Each trial began with placing a matador bug into the plexiglass cube alone for a baseline acclimation period of 30 min. We subsequently added either the praying mantid or the katydid based on preassigned order. Each treatment lasted for 30 min, after which time the stimulus arthropod was removed. After each trial, we wiped the box down with ethanol to eliminate any potential chemical signals before introducing the next matador individual. After completing the treatments, we moved the matador bug to a new butterfly cage to prevent interaction with individuals that had yet to be tested. A video camera (Canon R7, RF35 mm, 120 fps at 1,920 × 1,080 pixel) was positioned ∼50 cm from the plexiglass box, perpendicular to its front face, ensuring that the entire box was in frame from a side-on view.

### Across species data collection

We aimed to document flag-waving behavior in other species of coreid bugs, through direct observation of local species found in Panamá and by a broad general search of videos online. We used the key words “coreid,” “bug,” “waving,” “anisoscelis” (as all searches were done prior to the genus name change) in different combinations as search terms in YouTube to search for videos. We labeled an insect as “flag-waving” when insects lifted their hind legs and oscillated their tibias, similar to the behavior we saw in matador bugs. Only videos where species could be clearly identified and where location was documented were added to our list. Although our methods likely underestimate the number of species in this group that perform this distinctive behavior, our aim was to add to our knowledge of the commonness of this behavior among species with similar morphologies.

### Behavioral analysis

We reviewed all of our recorded video footage from our experimental setup using VLC media player and recorded the waving frequency (number of waves in total), waving intensity (the number of waves per bout), and wave duration (the total time spent waving). We also recorded instances when the praying mantid attacked the bug and the location on the bug that the attack was targeted. We defined an individual wave as a single occurrence of 1 tibial flag being raised above the body (sensu Longbottom et al. 2022) A discrete waving bout was defined as starting when the flag was first lifted and ending when the leg was lowered and remained stationary for at least 2 s (sensu Longbottom et al. 2022). Within each bout, the count of individual waves was recorded. The duration of waving was calculated from the difference between the start and the end times of these discrete bouts. Waving bouts varied in duration, ranging from short instances consisting of a single flag wave (<1 s) to prolonged bouts encompassing more than 45 waves (>1.5 min) (see Supplementary S3, Video S2). In the few instances where mantids attacked matador bugs, we also recorded the time of each praying mantid attack, noting whether the attack occurred during a waving bout or when the bug was not waving. In addition, we recorded the time of the last wave before an attack, as well as the region of the matador body attacked.

### Data analysis

**Figure 2:**
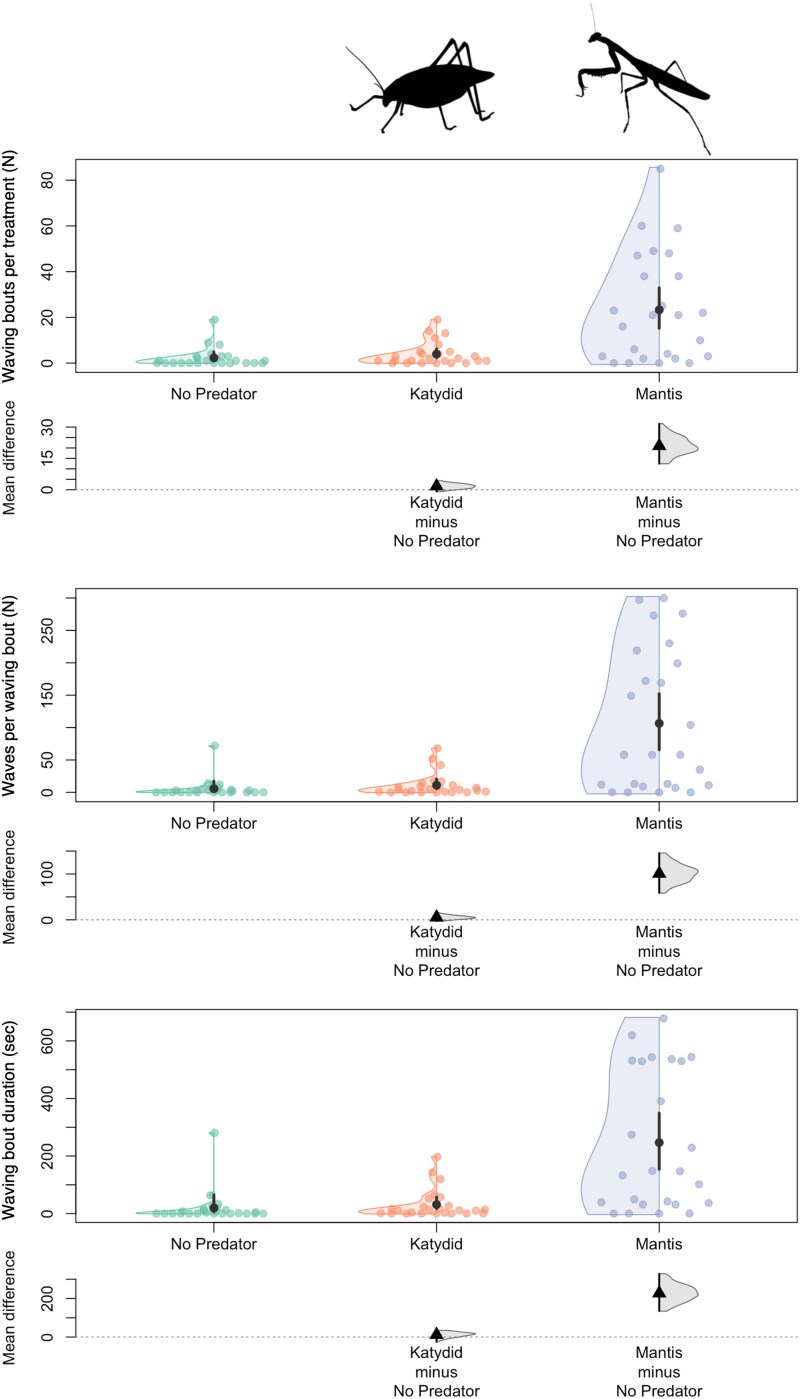
Flag-waving behavior in matador bugs (*Bitta alipes*) in response to 3 treatments: no predator (baseline), a katydid (nonpredator), and a praying mantid (predator). The panels show the number of waving bouts (top panel), the number of leg waves per bout (middle panel), and the duration of each bout in seconds (bottom panel). The presence of a praying mantid significantly increased waving behavior compared with both the katydid and the no-predator controls, resulting in approximately a 7-fold increase in the number of bouts and a 1.5-fold increase in both waves per bout and bout duration. The top panels show raw data with means ± SE, whereas the bottom panels display Hedges’ *g* effect sizes calculated as mean differences from the no-predator baseline (Khan and McLean 2024). Data represent 2,938 individual leg waves clustered into 681 bouts from 25 matador bugs.

We used generalized linear mixed-effects models to analyze the effect of different treatment conditions on the waving behavior of matador bugs. Given that our data exhibited overdispersion, where the variance exceeds the mean, we used a negative binomial distribution for 2 response variables: the number of waving bouts (frequency) and the number of waves per bout (intensity), as this distribution is more appropriate than a standard Poisson model (Hilbe 2011). Because the duration of waving (time) was a continuous variable with a positively skewed distribution, we used a Gamma model for this response variable. Each global model included the following fixed effects: Stimulus (nonpredatory katydid or praying mantid), sex of the matador bug, pronotum width of the matador bug, pronotum width of the stimulus (katydid or mantid), the order in which the matador bug was exposed to the stimulus (whether it faced the mantid or katydid first) as predictor variables, and lastly a trial set was included to account for the nonrandomized order of the first 18 trials. The trial order allowed us to test whether prior experience with 1 type of insect makes matador bugs more or less reactive to another insect. In addition, the trial set allowed us to test the effects of the consecutive order, relative to a random order (i.e., mantis first katydid or randomized order) for each set of trials that occurred over a range of times. To account for the nonindependence of multiple behavioral measurements from the same matador individual, we included a random intercept for each bug. We ran a variance inflation factor (*vif*) test to determine whether any of these parameters were multicollinear and found significant multicollinearity between the stimulus order and the trial set and thus we removed the trial set from all models (see Supplementary S4 for full model details). We fit negative binomial models using the “glmer.nb” function from the “lme4” package in R (Bates et al. 2015) and implemented the Gamma model using the “glmer” function. We checked the normality of our data by visually inspecting histograms. To assess the model fit, we checked residuals’ plots and our negative binomial models for overdispersion. We visually depicted our results by calculating Hedge's *g* effect sizes (Khan and McLean 2024) for both predatory and nonpredatory treatments, using our acclimation period as a baseline for comparisons (Fig. 2).

## Results

### Behavior

In 25 h of experimental observation, we documented 2,938 individual leg waves clustered into 681 bouts from 25 matador bugs (males: 12, females: 13). The total observation time was divided into 2 treatment conditions, each lasting 30 min per bug: nonpredatory katydid and praying mantid. The mean number of waving bouts varied across treatment conditions, with the nonpredatory katydid treatment showing a mean of 2.19 bouts (variance = 2.47), and the praying mantid treatment showing a mean of 3.65 bouts (variance = 15.63). The variance exceeded the mean in both conditions, indicating overdispersion, particularly in the mantid treatment.

Our statistical analyses revealed that matador bugs increased their waving behavior in the presence of a praying mantid compared with the nonpredatory katydid. Specifically, our model with treatment included as a fixed effect revealed that the mantid treatment had a statistically significant (*P* < 0.05) effect on the frequency of bouts (estimate = 1.77, SE = 0.108, *z* = 16.42, *P* < 0.01), the intensity of bouts (i.e., number of waves; estimate = 0.45, SE = 0.10, *z* = 4.48, *P* < 0.01), and the duration of bouts (estimate = 0.31, SE = 0.11, *z* = 2.88, *P* < 0.01). The estimates indicate that the presence of a praying mantid corresponds to a roughly 7-fold increase in the number of waving bouts and a 1.5-fold increase in both the number of waves per bout and the duration of each bout (Fig. 2). Additionally, the highest wave count in a single bout for the mantid treatment was 46 with waves, lasting 103 s, compared with the katydid treatment where the highest wave count was 10 waves, lasting 31 s. Notably, in the mantid treatment, there were 54 bouts with more than 10 waves and 36 bouts that lasted longer than 31 s, whereas in the katydid treatment, no bouts exceeded these counts or durations.

None of the additional predictor variables—sex of the matador bug, pronotum width of the matador bug, pronotum width of the stimulus (katydid or mantid), the order in which the matador bug was exposed to the stimulus (whether it faced the mantid or katydid first)—had a significant effect on any of the 3 response variables: the number of waving bouts (frequency), waves per bout (intensity), or waving bout duration (time). Given this lack of significance in the other parameters and to avoid overparameterizing our relatively small dataset, we report our statistics from pared-down versions of the models with only treatment included as a fixed effect and bug ID included as a random effect. Detailed reports of these analyses can be found in the Supplementary Materials; code and data sheets can be found on the Figshare link supplied below.

Praying mantids performed a total of 7 predatory attacks on the bugs: 4 were unsuccessful, where the bug successfully evaded capture, and 3 were successful, wherein the mantid captured and consumed the matador bug. One mantid consumed 1 matador bug, and another mantid consumed 2 bugs- thus given that only 2 mantids consumed bugs in total, we did not have sufficient data to test whether mantids are less likely to attack a bug after having eaten 1. Of 7 total attacks, 2 were directed at the tibial flags, whereas the remaining 5 targeted the main body (thorax/abdomen). The 3 matador bugs that were attacked and predated did not exhibit any waving behavior for at least 57 s prior to the attack and were captured within the first 5 min of the trial.

### Across species results

In addition to the matador bug, we found an additional 5 species that perform similar elaborate waving behavior. Two species of coreid locally in Panama: *Diactor bilineatus* and *Bitta gradadia* (Somjee U, personal observation). We also found an additional 3 species from online videos that display elaborate flag-waving behavior, which include *Bitta lurida*, *Anisoscelis foliaceus*, *Bitta hymeniphera* (see Supplementary Materials). However, we also found other species that have less conspicuously colored expanded tibia in the genera *Leptoglossus* and *Acanthocephala*, which do lift a hind leg, but we found no evidence that they perform elaborate flag waving behavior.

## Discussion

This study supports the hypothesis that the matador bug's flag-waving behavior is a targeted response to the presence of an arthropod predator. Waving bouts increased 7-fold in the presence of a mantid compared with a nonpredatory katydid, consistent with the idea that waving behavior acts as a specific antipredation strategy rather than a general reaction to proximate stimuli (Figs. 2 and S2). Notably, the highest wave count in a single bout during the katydid treatment was 10 waves, lasting 31 s, whereas 54 bouts in the mantid treatment exceeded this count. This increased frequency, intensity, and time-performing waving behavior in the presence of a mantid supports the idea that this waving behavior may act as an adaptive response to predators. Moreover, on multiple occasions, the matador bug remained relatively unresponsive to the katydid (see Supplementary S3, Video S3). Our analysis revealed no effect of sex on waving behavior, consistent with previous findings (Longbottom et al. 2022). Additionally, the order in which the matador bug encountered either stimulus (predator or nonpredator first) had no effect on waving behavior, suggesting that this behavior is displayed in response to an active threat. This indicates that the matador bugs perceived the katydid as a nonthreatening stimulus, further supporting the hypothesis that waving is a targeted antipredator defense rather than a generalized response to other arthropods. However, it is likely that waving behavior is deployed more in response to smaller predators that have search images at closer range such as small vertebrates (e.g., lizards and frogs), in addition to predatory arthropods, and future studies are needed to examine this hypothesis.

Matador bugs were attacked by mantids a total of only 7/25 times, and 3 of these attacks led to a consumption of the bug, with 1 mantid consuming 2 individuals. Notably, all of the attacks occurred when individuals were not actively waving, and in each case, the head, pronotum, and tibial flags were rejected. Previous work has shown these bugs to be relatively unpalatable to avian predators (Rubin et al. 2024). Although palatability trials have not been conducted between matador bugs and mantids, previous studies have tested taste aversion learning in mantids and found that they will learn to avoid experimentally created bitter-tasting prey (Carle et al. 2018 ), as well as truly chemically defended prey (Bowdish and Bultman 1993; Prudic et al. 2007). They have also been shown, similar to many other animals, to reject unpalatable body parts of prey while consuming palatable parts (Rafter et al. 2013; Mebs et al. 2017; Tarvin et al. 2023). Along these lines, in the motmot-bug study, motmots that attacked palatable crickets with matador bug flag legs experimentally added removed the flags 93% of the time before ingesting the cricket (Rubin et al. 2024), although it is also possible that legs were removed to make the insects easier to swallow. More studies with repeated encounters between matador bugs and invertebrate predators is needed to know whether the waving behavior facilitates learning about bug chemical defense, especially for predators that are less sensitive to typically aposematic colors (e.g., red and orange) (Svádová et al. 2009; Skelhorn et al. 2016).

**Figure 3:**
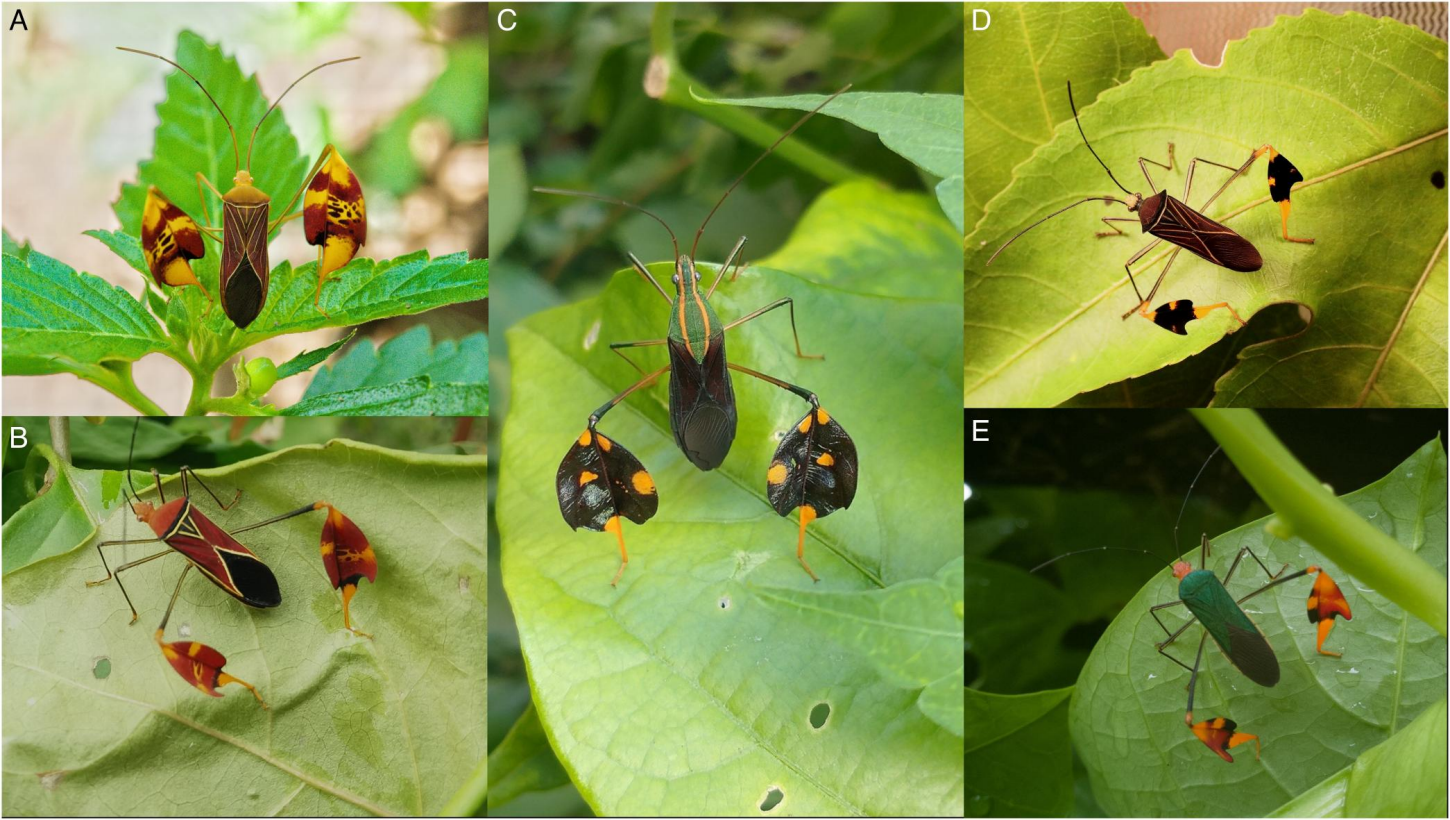
Elaborate flag-waving behavior is found in at least 6 species of coreid bugs with tibial flags. All species that display this behavior possess conspicuous, elaborate tibial flags and are *Passiflora* specialists. Flag waving is found in *Bitta alipes* (Fig. 1), and in (A) *Bitta lurida*, (B) *Bitta hymeniphera*, (C) *Diactor bilineatus*, (D) *Bitta gradadia* and (E) *Anisoscelis foliaceus*. Photo credits: *B. lurida* © adel-fridus, iNaturalist observation: https://www.inaturalist.org/observations/249183515, accessed 17 March 2025; *B. hymeniphera* © Erika Nathaly Bernal Morales, iNaturalist observation: https://www.inaturalist.org/observations/48812802, accessed 17 March 2025; *D. bilineatus* © tyski, 2023, iNaturalist observation: https://www.inaturalist.org/observations/188839210, accessed 17 March 2025; *B. gradadia* photo by Ummat Somjee; *A. foliaceus* © Cristian Serrano, iNaturalist observation: https://www.inaturalist.org/observations/54789401, accessed 17 March 2025 (License: CC BY-NC 4.0).

In addition to the matador bugs, we found elaborate tibial flag displays in 5 other related coreid species that feed on *Passiflora* (Fig. 3). It is important to note that other species of coreid that have enlarged femur muscles and rigid tibial plates and are used in intraspecific combat have not been reported to perform the elaborate waving behavior (Eberhard 1998). Many coreid species have elaborated leg traits, yet most studies have focused on their role as weapons in sexual selection. Larger femoral muscles function together with tibial spines and/or rigid tibial expansions (tibial plates) to squeeze opponents during combat (Mitchell 1980; Miyatake 1997; Eberhard 1998; Tatarnic and Spence 2013). Elaborated hind leg morphology that functions in combat and intraspecific signaling is often associated with a metabolically costly muscle (Somjee et al. 2017, 2018; O’Brien et al. 2019; Somjee 2021); such femora muscle appears reduced in matador bugs and other flag-waving bugs. Examples of coreid species with such intraspecific competitive morphology such as large femora muscles and tibial plates are found in the genera *Acanthocephala*, *Leptoglossus*, and *Narnia.* These genera typically have positive allometry of their hind-leg traits and will sometimes raise their hind legs in a threat posture in response to conspecifics (Mitchell 1980; Miyatake 1997; Eberhard 1998; Procter et al. 2012, Supplementary Video). Their tibial expansions, however, are typically less conspicuously colored compared with the “flag-waving” species described here. *Passiflora* is generally considered to be unpalatable, due to its own chemical defense, yet every assayed insect species that does feed on this plant has been found to assimilate cyanogenic compounds, and most display aposematic coloration to advertise this defense (Pinheiro De Castro et al. 2020). It is likely that the matador bug assimilates cyanogenic compounds (Aldrich 1988), but future studies are needed to specifically test this hypothesis. We note that all the coreid species that we found to use elaborate flag-waving behavior also have elaborate tibial flags with conspicuous color patterns (Fig. 3). It is also notable that *Holymenia histro*, a closely related species to the matador bug, does not possess elaborate flags and does not perform waving behavior. However, this species is a wasp mimic with clear hemelytra and “wasp-like” antennal movement that likely enhance this effect; thus, this may represent a different avenue to antipredator avoidance (Rodrigues et al. 2005; Pereira et al. 2013). These findings are consistent with the idea that some *Passiflora*-feeding coreid bugs have evolved elaborate hindleg flags and waving behavior that communicate chemical defense to predators, but different coreids have established different antipredator behavior and morphology such as wasp mimicry. Studies with these other species are required to test this idea more broadly in the group.

This work opens up many new questions about how predator-specific behaviors function to dissuade predation. The waving behavior of the matador bug serves as a deterrent signal to predators, as suggested by the absence of mantid attacks during this behavior. Because the mantids used in this study were sympatric, it is plausible that they had previously encountered matador bugs and learned to associate waving with unprofitability. The waving behavior may signal that the matador bug is prepared to flee (Longbottom et al. 2022), similar to the stotting behavior seen in Thomson's gazelles, which conveys readiness to escape (Caro 1986; FitzGibbon and Fanshawe 1988). Additionally, flag waving may be an aposematic behavioral signal to invertebrate predators, such as the praying mantid, which would not be able to detect the bug's aposematic coloration (Fabricant and Herberstein 2015). Alternatively, this behavior could be an intimidation strategy, used to make the bug appear larger and more threatening to an approaching predator. A set of foundational neurobiology studies using frogs and toads demonstrated that a square moving toward the anuran from above was perceived as a threatening object (Ingle and Hoff 1990), causing the animal to move away. Given the shape and size of the matador bug's flags, especially in comparison with the size of the mantid itself, it is possible that the downward sweep of the flags during waving behavior elicits an innate fear response in mantids and other arthropod predators. More studies are needed to see whether flag waving ever leads to a flight response in the putative predator.

Although not directly tested, our study found little evidence for the deflection hypothesis: no attacks occurred while matador bugs were waving, and only 7 total attacks were recorded for more than 12.5 h of mantid exposure. Although 2 of these attacks were directed toward the hind flags of the matador bug (see Supplementary S3, Video S4), we cannot definitively say that mantids perceived these flags as alternate targets. Previous research has indicated that the hind flags may not deflect strikes from chicks (Rubin et al. 2024), suggesting that the waving behavior may serve a different function. Nonetheless, the deflection hypothesis requires more precise studies (Hendrick et al. 2022). For example, a video from the Longbottom et al. (2022) study shows a jumping spider attacking the flag of a matador bug during waving, suggesting that this behavior may create a visual decoy for some predators (see Supplementary S3, Video S5). We suggest that the deflection hypothesis in the matador bug warrants further investigation, particularly by using jumping spiders, as they are known predators of many coreids (Somjee U, personal observation). The matador bug has among the most rapid autotomy in coreids, and a relatively large number of individuals observed in the wild are missing at least 1 hind leg, consistent with the idea that waving behavior may serve a deflective function against some predators (Emberts et al. 2020; Longbottom et al. 2022).

We note a few caveats to this study. Although we used 25 individual matador bugs, the number of individual mantids, which consisted of different, but size-matched species, was relatively low (*n* = 7), as was the number of nonpredatory katydid individuals (*n* = 3). The experiments also took place in an unnaturally simplified environment, which may have affected the detection rate of both predator and prey to each other and, consequently, increased different aspects of waving by the matador bug. In addition, our study used mantids and katydids from different species, and although we found a strong effect of predator (mantid) versus nonpredator (katydid) on waving behavior, future studies should examine potential species-specific responses. In general, further research is needed to understand how these interactions would occur in a more ecologically-relevant environment with other invertebrate predators.

We have tested for the first time the role of waving behavior in matador bugs in the context of predation, finding that this elaborate behavior is likely a response to the identification of a nearby predator. The matador bug's waving behavior, combined with its aposematic coloration and rapid autotomy, provides opportunities to examine how diverse antipredation strategies may work synergistically to deter different predator types (Kikuchi et al. 2023). Our study has broader implications for understanding the evolution of facultative antipredator traits. Future studies should explore the visual cues that invertebrate animals, such as matador bugs, use to detect and selectively deploy antipredator traits against specific predator types. Investigating the evolutionary correlation between elaborate antipredator traits and predator recognition will contribute to our understanding of how predator-specific pressures drive the evolution of multifunctional conspicuous behaviors in prey species.

## Supplementary Material

zoaf047_Supplementary_Data

## Data Availability

All the data used for statistical analysis is available at figshare: https://figshare.com/s/a0bc34cbe9e2b7046196?file=53301608. Videos of flag waving behavior can be found: https://www.youtube.com/playlist?list=PLiJkt–i.
