# Supplementary material for: Flag-waving behavior in matador bugs is an antipredatory strategy
Source: Curr Zool. 2025 Aug 1;72(2):293–302. doi: 10.1093/cz/zoaf047 (PMC13202313; doi:10.1093/cz/zoaf047)
Supplement: zoaf047_Supplementary_Data [file zoaf047_supplementary_data.zip › Supplementary_Materials_Revised.docx]

# Supplement: Flag-waving behavior in matador bugs is an anti-predatory strategy

#
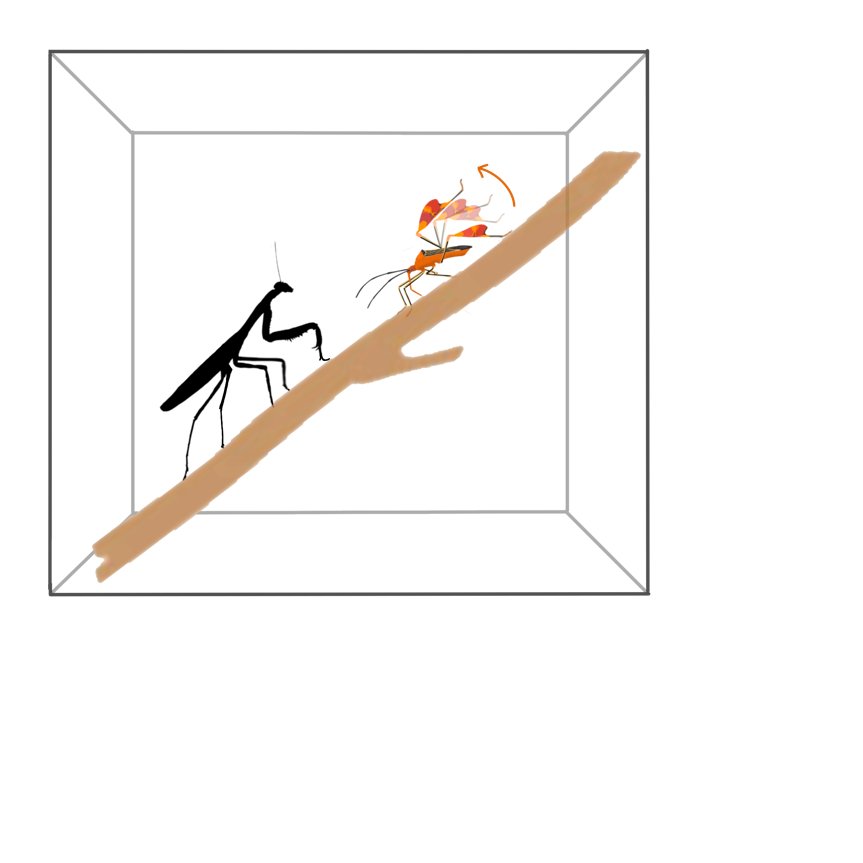
Figure S1

Figure S1 - Experimental setup showing a matador bug (Bitta alipes) inside the plexiglass cube (15×15×15 cm) with a natural twig perch. The bug is illustrated performing a flag-waving display, which was recorded during exposure to either a predator (praying mantid) or non-predator (katydid).

# Figure S2

#

Figure S2: The matador bug exhibits low-frequency flag-waving behaviour in the absence of external stimuli and shows no increase in response to conspecific males or females (Longbottom et al. 2022). In this study, we found that flag-waving rate increased significantly in response to a predatory arthropod (mantid), but not in response to a non-predatory arthropod (katydid).

# S3 - Experimental Videos

# Videos of experiment and flag-waving behavior 1:4 can be found here:

<https://www.dropbox.com/scl/fo/5drraeb1f3hzwqa1394ft/AH5s0p2yiq7ZhkvpEd6mldI?rlkey=q80e7t3nbyntlmokbfz8lfmy7&st=xg1hl458&dl=0>

# S4

# 1. Detailed model outputs

We used generalized linear mixed models (GLMMs) to test the effect of treatment (mantid vs katydid) and covariates on three measures of matador bug waving behavior: number of bouts, waves per bout, and bout duration. All models included the fixed effects of treatment, sex of the matador bug, pronotum width of the matador bug, pronotum width of the stimulus arthropod, trial set (a, b, or c), and whether the mantid was presented first. Bug identity was included as a random intercept. Models were fitted using the lme4 package in R.

## 1.1 Global Model: Number of Waving Bouts (Frequency)

Model formula:
glmer.nb(Bouts ~ Treatment + Sex + Bug_Pronotum_Width + Stimulus_Pronotum_Width + Trial_Set + Mantid_First + (1|Bug), data = kmg)

Model fit statistics:
- Family: Negative Binomial (θ = 79.31)
- AIC = 336.4, BIC = 354.7, LogLik = -158.2
- Observations: 46, Groups: Bug (n = 23)

| **Predictor** | **Estimate** | **Std. Error** | ***z*-value** | ***P*-value** |
| --- | --- | --- | --- | --- |
| (Intercept) | -3.44 | 6.33 | -0.54 | 0.587 |
| Treatment (Mantid) | 1.93 | 0.15 | 13.21 | <0.001 |
| Sex (Male) | 0.94 | 0.67 | 1.42 | 0.157 |
| Bug Pronotum Width | 0.62 | 1.25 | 0.49 | 0.621 |
| Stimulus Pronotum Width | 0.04 | 0.06 | 0.69 | 0.494 |
| Trial Set b | -1.79 | 1.27 | -1.41 | 0.158 |
| Trial Set c | -0.13 | 1.00 | -0.13 | 0.898 |
| Mantid First | 1.60 | 1.02 | 1.57 | 0.116 |

## 1.2 Global Model: Waves per Bout (Intensity)

Model formula:
glmer.nb(Wave_number ~ Treatment + Sex + Bug_Pronotum_Width + Stimulus_Pronotum_Width + Trial_Set + Mantid_First + (1|Bug), data = kmr)

Model fit statistics:
- Family: Negative Binomial (θ = 2.58)
- AIC = 3194.6, BIC = 3239.3, LogLik = -1587.3
- Observations: 646, Groups: Bug (n = 20)

| **Predictor** | **Estimate** | **Std. Error** | **z-value** | ***P* -value** |
| --- | --- | --- | --- | --- |
| (Intercept) | 1.01 | 1.39 | 0.73 | 0.465 |
| Treatment (Mantid) | 0.44 | 0.12 | 3.74 | <0.001 |
| Sex (Male) | 0.05 | 0.12 | 0.41 | 0.680 |
| Bug Pronotum Width | 0.00 | 0.25 | -0.01 | 0.989 |
| Stimulus Pronotum Width | -0.03 | 0.04 | -0.68 | 0.496 |
| Trial Set b | 0.32 | 0.26 | 1.24 | 0.216 |
| Trial Set c | 0.28 | 0.22 | 1.29 | 0.197 |
| Mantid First | 0.02 | 0.21 | 0.09 | 0.928 |

##

## 1.3 Global Model: Waving Bout Duration (Time)

Model formula:
glmer(Waving_bout_duration ~ Treatment + Sex + Bug_Pronotum_Width + Stimulus_Pronotum_Width + Trial_Set + Mantid_First + (1 | Bug), data = kmr, family = Gamma(link = "log"))

Model fit statistics:
- Family: Gamma (log link)
- AIC = 4303.8, BIC = 4348.5, LogLik = -2141.9
- Observations: 646, Groups: Bug (n = 20)

| **Predictor** | **Estimate** | **Std. Error** | **t-value** | ***P* -value** |
| --- | --- | --- | --- | --- |
| (Intercept) | 3.07 | 1.52 | 2.02 | 0.044 |
| Treatment (Mantid) | 0.28 | 0.13 | 2.16 | 0.031 |
| Sex (Male) | 0.01 | 0.13 | 0.05 | 0.963 |
| Bug Pronotum Width | -0.15 | 0.28 | -0.55 | 0.580 |
| Stimulus Pronotum Width | -0.05 | 0.04 | -1.20 | 0.230 |
| Trial Set b | 0.18 | 0.29 | 0.64 | 0.520 |
| Trial Set c | 0.33 | 0.24 | 1.36 | 0.175 |
| Mantid First | -0.12 | 0.24 | -0.50 | 0.616 |

**1.4 Simple Model: Number of Waving Bouts (Frequency)**

glmer.nb( Bouts ~ Treatment + (1 | Bug), data = kmr, family = Gamma(link = "log"))

| **Predictor** | **Estimate** | **Std. Error** | **t-value** | ***P* -value** |
| --- | --- | --- | --- | --- |
| (Intercept) | 1.3762 | 0.2726 | 5.049 | 4.44e-07 |
| Treatment (Mantid) | 1.7713 | 0.3745 | 4.731 | 2.24e-06 |

## 1.5 Simple Model: Waves per Bout (Intensity)

glmer.nb(Wave_number ~ Treatment + (1 | Bug), data = kmr, family = Gamma(link = "log"))

| **Predictor** | **Estimate** | **Std. Error** | **t-value** | ***P* -value** |
| --- | --- | --- | --- | --- |
| (Intercept) | 1.0257 | 0.1047 | 9.798 | < 2e-16 *** |
| Treatment (Mantid) | 0.4488 | 0.1002 | 4.477 | 7.56e-06 *** |

## 1.6 Global Model: Waving Bout Duration (Time)

glmer( Waving_bout_duration ~ Treatment + (1 | Bug))

| **Predictor** | **Estimate** | **Std. Error** | **t-value** | ***P* -value** |
| --- | --- | --- | --- | --- |
| (Intercept) | 2.0466 | 0.1103 | 18.561 | < 2e-16 *** |
| Treatment (Mantid) | 0.3127 | 0.1087 | 2.876 | 0.00403 ** |

# S5. Details of mantis attacks

Matador bug #4 was attacked once by mantis #2, with the strike targeting the body, resulting in successful capture. Matador bug #11 faced two attacks from mantis #1; the first attack targeted the flag and was unsuccessful, while the second strike targeted the body and was successful. Matador bug #18 was successfully captured with a strike to the body from mantis #1. Matador bug #19 encountered an unsuccessful attack from mantis #1 directed at the body. Matador bug #25 faced two attacks from mantis #1; the first was directed at the flag and the second at the body, both of which were unsuccessful.

#

# S6. Repeatability analysis

To assess and minimize measurement error, we remeasured the data and conducted a repeatability analysis. Specifically, the same observer remeasured the total number of waves in every 10th bout from the dataset. A linear model was then constructed, regressing the initial measurements against the remeasurements. The higher the coefficient of determination (R²) between the initial measurement and the second measurement would be consistent with a lower incidence of measurement error and a higher confidence in our measurements.

Our linear model that regressed the first measurements against the remeasurements of the wave count in every 10th bout, resulting in a total of 76 remeasurements, showed a very high correlation (b = 0.986, R² = 0.994), indicating strong repeatability and minimal measurement error. This increases our confidence that our initial measurements of flag-waving behavior were consistent (Fig. 1).


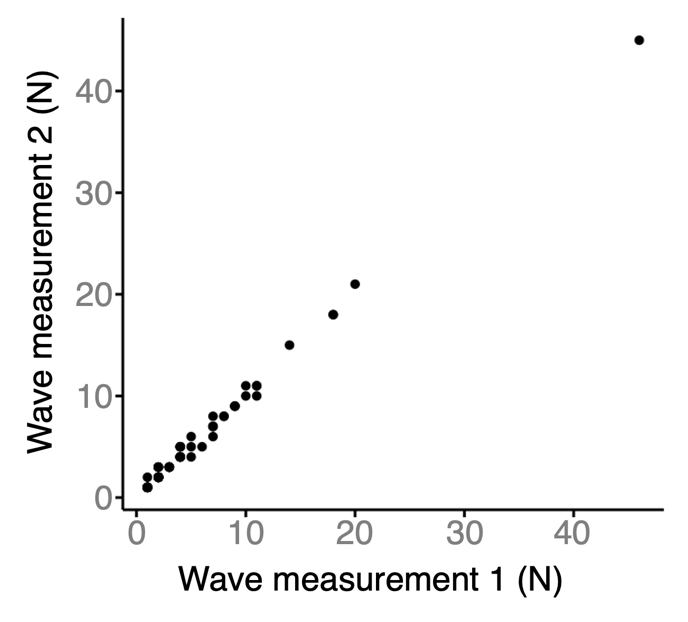


*Figure S6:* *Repeatability of wave counts from matador bugs (Bitta alipes), based on remeasurement of every 10th bout. Wave measurements 1 and 2 represent the original counts and remeasurements, respectively. A high correlation (b = 0.986, R² = 0.994) indicates strong repeatability and minimal measurement error. Data represents 76 remeasurements.*

# S7. Across species videos

1. *Bitta lurida*: <https://youtu.be/mZ3xFGvGVk0>. @yelapanature4336. 2016. Yelapa, Mexico.
2. *Anisoscelis foliaceus:* <https://youtu.be/xws_Y3I_ynI>. @gravandoavida. 2024. Brazil
3. *Bitta hymeniphera:* (0:27 Seconds): <https://youtu.be/Mmo2agKWKYE?t=27>. @thewildlifemaster5300. 2022. Tobago.
4. *Diactor bilineatus*: <https://youtu.be/mUPjZHJ_du4>. @ducampos8601 .2024. Brazil.
5. *Leptoglossus* sp.– antipredator leg raise: <https://youtu.be/k9Sto-xXI6k>. @insectsandanimals5535. 2021.Mexico.
